# Transformation of a low‐grade glioma into a glioblastoma along with the development of lung and mediastinal lymph node metastases after repeated craniotomy: A case report

**DOI:** 10.1002/ibra.12119

**Published:** 2023-07-06

**Authors:** Yunan Wang, Hua Yang, Jun Su, Xiaobin Jian, Peijie Li, Jianguo Zhou, Wei Hu

**Affiliations:** ^1^ Department of Thoracic Oncology The Second Affiliated Hospital of ZunYi Medical University Zunyi Guizhou China; ^2^ Department of Pathology The Second Affiliated Hospital of ZunYi Medical University Zunyi Guizhou China; ^3^ Department of Pathology The Affiliated Hospital of ZunYi Medical University Zunyi Guizhou China

**Keywords:** extracranial metastasis, glioblastoma, low‐grade glioma, lung, malignant transformation

## Abstract

Extracranial metastasis of glioma is extremely rare. Herein, we report a case of glioblastoma that originated and showed stepwise malignant transformation from a low‐grade glioma (LGG) along with the presence of lung and mediastinal lymph node metastases after repeated craniotomy. A 30‐year‐old man presented with hemoptysis. Thoracic computed tomography revealed a space‐occupying lesion in the right upper lung with mediastinal nodal and metastases in both lungs; lung cancer was suspected. The patient's medical history showed that he had undergone craniotomy three times in 7 years for a primary LGG disease relapse, and stepwise malignant‐transformed high‐grade glioma (HGG). However, brain magnetic resonance imaging did not reveal any relapse of intracranial tumors. The diagnosis of extracranial metastatic glioblastoma was confirmed using the morphology and staining results for specific immunohistochemistry markers using the specimen obtained via endobronchial ultrasound transbronchial needle aspiration. Subsequently, the patient received a combination of systemic and local treatments; however, he died of massive hemoptysis after 6 months. The survival time of this glioma patient improved after transformation and metastasis. Detailed descriptions will help us understand the biological behavior of glioma, but more studies are needed to confirm the complex mechanism of extracranial metastasis.

## Graphical abstract

A male patient suffered from recurrent primary low‐grade glioma (LGG) disease and progressive transformation to high‐grade glioma (HGG) within 7 years, and underwent 3 craniotomy operations. However, brain magnetic resonance imaging did not reveal intracranial tumor recurrence. Extracranial metastatic glioblastoma was confirmed by morphology and specific immunohistochemical marker staining after a bronchial ultrasound and bronchial needle sampling.

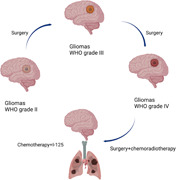

## 1 INTRODUCTION

Gliomas are the most common primary tumors in the central nervous system, accounting for more than half of all cases of intracranial tumors. Low‐grade gliomas (LGGs) comprise a heterogeneous group of tumors with varying patient survival. A considerable proportion of LGG cases may transform into high‐grade gliomas (HGGs) during that duration.^1^, ^2^, ^3^, ^4^ However, extraneural metastasis of glioma is extremely rare, even metastasis from the most aggressive tumor type, that is, glioblastoma.^5^, ^6^ Therefore, once metastasis is observed, it is important to distinguish it from second primary tumors at metastatic sites. Herein, we present the diagnosis and treatment of an extraneural metastatic glioblastoma that originated from a stepwise malignant‐transformed LGG after repeated craniotomy without the observation of intracranial relapse, as such cases could be easily confused with primary lung cancer.

## 2 CASE PRESENTATION

**Figure 1:**
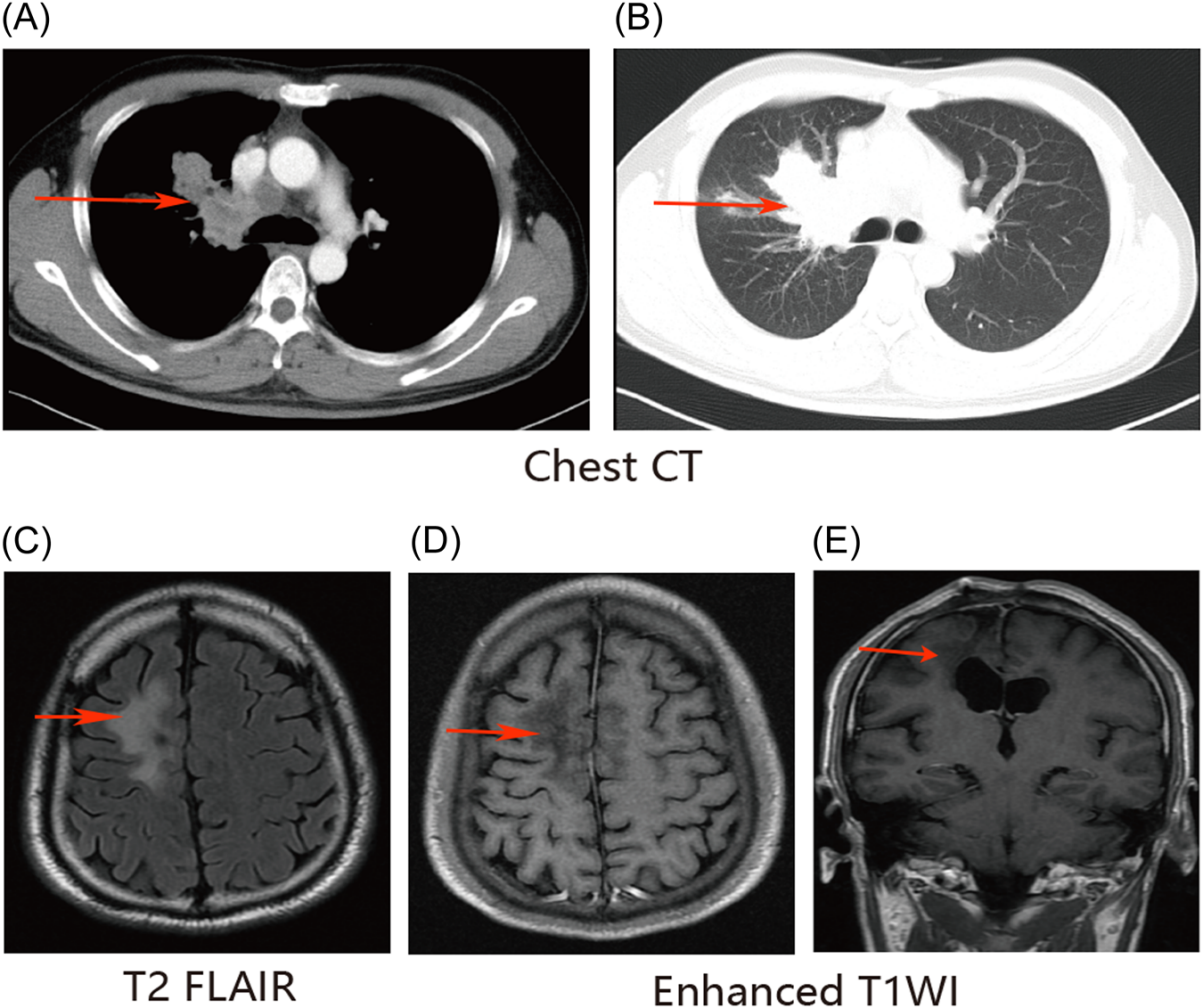
Results of computed tomography (CT) examination of lung and magnetic resonance imaging (MRI) examination of brain of patient when first seeing a doctor for hemoptysis (September 2016). CT examination of lung or MRI examination of brain, and arrows point to the lesion. [Color figure can be viewed at wileyonlinelibrary.com]

**Figure 2:**
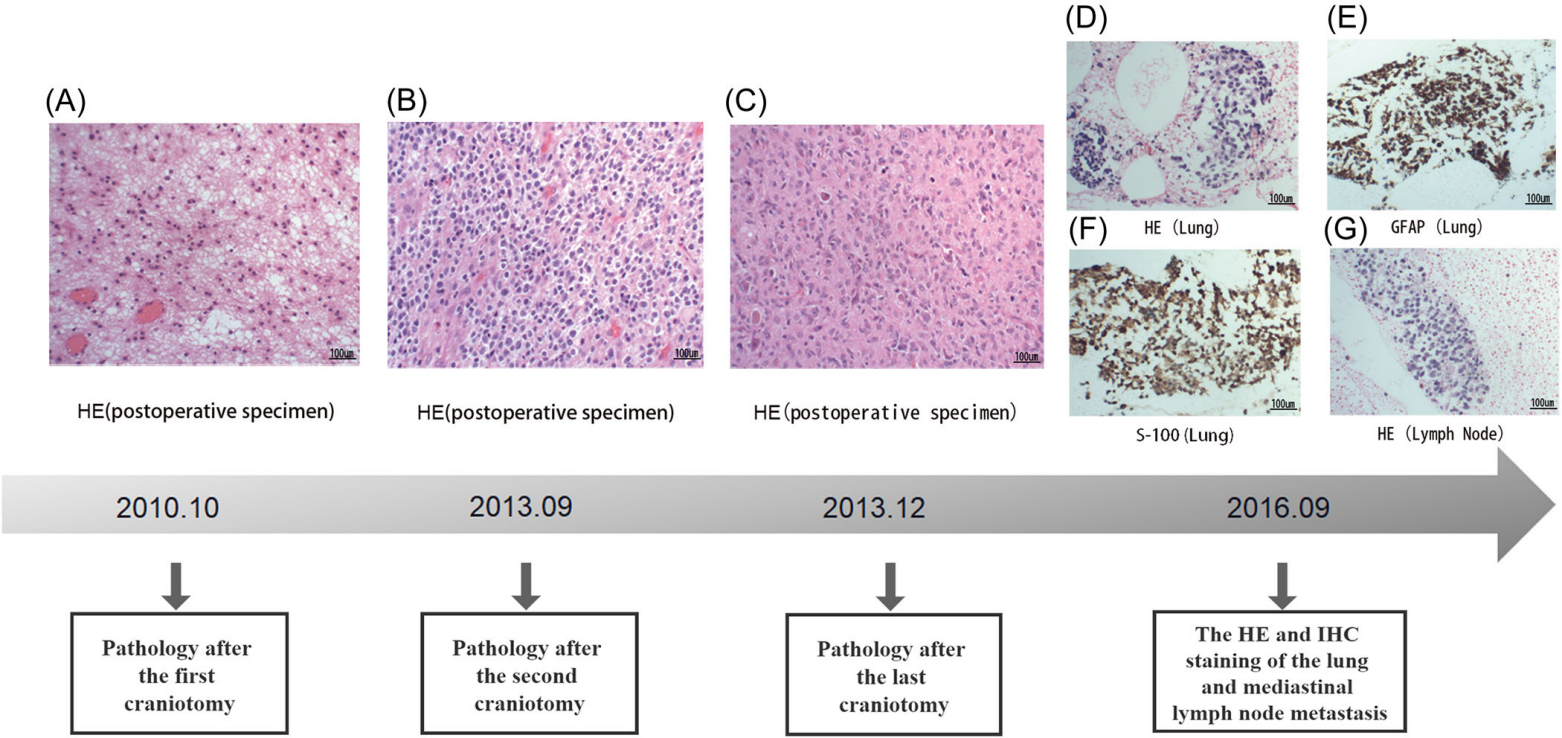
Relevant pathological examination results after multiple operations and invasive examinations. HE, Hematoxylin‐eosin staining. [Color figure can be viewed at wileyonlinelibrary.com]

A 30‐year‐old man visited the emergency department of our hospital for hemoptysis in September 2016. Thoracic computed tomography (CT) revealed a space‐occupying lesion in the right upper lung with mediastinal nodal and multiple metastases in both lungs; lung cancer was suspected (Figure 1A,B). After conservative hemostatic treatment, respiratory physicians obtained the following detailed medical history of the patient: the patient was initially diagnosed with a space‐occupying lesion in the right frontal lobe when he presented with repeated seizures 7 years ago in October 2010 at a local hospital. Then, the tumor was completely resected, and histologic analysis revealed a World Health Organization (WHO) grade II astrocytoma (Figure 2A). In September 2013, that is, 3 years later, the patient visited the local hospital again owing to weakness in the left lower limb, and magnetic resonance imaging (MRI) of the brain revealed relapse of the tumor in the frontal lobe (Figure S1A). After a second total resection (Figure S1B), histologic analysis revealed a WHO grade III astroglioma (Figure 2B); the polymerase chain reaction test indicated that the *O6 methyl guanine DNA methyltransferase* (*MGMT)* promoter was unmethylated, high‐resolution melting revealed that the *isocitrate dehydrogenase (IDH)* mutation was present, and fluorescence in situ hybridization (FISH) revealed that the 1p/19q deletion was negative. The local surgeon recommended postoperative adjuvant treatment, but the patient refused to continue treatment because all the symptoms resolved after surgery. However, after 3 months,i.e., in December 2013, the patient underwent the third craniotomy owing to weakness in the left lower limb (Figure S1C); the postoperative pathological analysis revealed a WHO grade IV glioblastoma (Figure 2C) while the results of the genetic tests were the same as those obtained previously. Subsequently, the patient received six cycles of chemotherapy with nimustine and cisplatin—according to the results of the drug‐sensitive test in vitro—and three dimensional‐chemoradiotherapy (total dose, 60 Gy/30 fractions). At the end of the treatment, the patient's symptoms improved significantly, with good quality of life. Since then, the patient was followed‐up irregularly.

On the basis of the medical history, extracranial metastatic glioblastoma was considered as a differential diagnosis; however, brain MRI revealed no evidence of intracranial tumor relapse (Figure 1C–E). Fiberoptic bronchoscopy was therefore performed for excluding the possibility of a second primary tumor in the lungs. No tumor was observed in the bronchial lumen. The histologic and immunohistochemistry analysis of the biopsy specimens from the lungs and mediastinal lymph nodes (Figure 2D,G), obtained via endobronchial ultrasound, showed that the tumor cell morphology was similar to that of glioblastoma; the tumor was positive for S100 and glial fibrillary acidic protein (GFAP) (effective markers of glioma; Figure 2E,F) and negative for thyroid transcription factor‐1 (TTF1) and P40 (markers of primary lung tumor). The diagnosis of extracranial metastatic glioblastoma was confirmed. To understand the metastatic route of the tumor, we performed the circulating tumor cell (CTC) test; however, we did not find CTCs of the glioblastoma in the peripheral blood. Subsequently, under the guidance of the multi‐disciplinary team in our hospital, the patient received two cycles of paclitaxel chemotherapy according to the drug sensitivity test in vitro. However, the disease could not be controlled. Therefore, crizotinib was subsequently administered to the patient according to the positive result of the *ALK* fusion gene test on FISH. Then, I‐125 seeds brachytherapy (ISB) was simultaneously implanted into the major sites of lung metastasis (Figure S2). There were no adverse effects during treatment. On February 15, 2017, the patient was hospitalized due to aggravation of cough and sputum with a small amount of bloodshot in the sputum. After symptomatic treatment such as anti‐infection, the patient was discharged with improvement. During the period, the patient still had hemoptysis problems. More than 1 month later, the patient was re‐admitted due to increased hemoptysis. After hemostasis and anti‐infection treatment, the patient got better. However, after 15 days, the patient died of massive hemoptysis in April 2017, and we were not able to evaluate the therapeutic effect of the administered treatment.

## 3 DISCUSSION AND CONCLUSION

Gliomas comprise 30% and 80% of primary brain tumors and malignant tumors, respectively.^7^ Malignant transformation was defined as the progression of a low‐grade tumor into a WHO grade III/IV tumor. Gliomas have the highest rate of malignant transformation among all tumors, HGGs have more aggressive biological behavior, the median survival time less than 15 months.^8^ In the current case, the malignant transformation of the glioma was undoubtedly the reason for metastasis. Age, tumor size, predominant histology of astrocytomas, neurologic symptoms, subtotal resection, and initial adjuvant therapy were associated with malignant transformation.^2^ However, in the current patient, there were no risk factors except for neurologic symptoms, predominant histology of astrocytoma, and the lack of adjuvant therapy at the initial stage. In a previous study by Murphy et al.,^1^ patient with *IDH* mutation combined with *MGMT* methylation and tumor protein P53 (TP53) mutation had significantly higher hazard ratios for malignant transformation. In the current case, the patient did not have any *IDH* mutation or *MGMT* methylation; however, it is unclear whether other genetic alternations might be involved in malignant transformation.

HGGs typically diffusely infiltrate the surrounding brain tissue. Extracranial metastasis is exceedingly rare and presents in no more than 2% of the cases.^9^ The motility of tumor cells and the breakdown of the blood–brain barrier were major factors of tumor cells disseminating into blood circulation. A previous meta‐analysis^5^ showed that patients who underwent open surgery accounted for a higher proportion of those with extracranial metastasis than those who underwent biopsy did. In the current case, metastasis occurred after repeated craniotomy, we believe that the repeated breakdown of the blood–brain barrier promoted extracranial colonization. Jian et al. showed that exosomes are a novel means of intercellular communication that crosses the blood‐brain barrier to regulate glioma invasion, and immune escape, and may become new biomarkers.^10^

The detection of CTCs is an effective method to understand the mechanism of metastasis of tumor cells through blood circulation.^5^ However, it is unclear why we did not observe any CTCs in the blood, although the absence of CTCs may be associated with the time of detection. Considering the medical history, we speculate that malignant‐transformed glioma cells had disseminated into the blood circulation after the last surgery and settled in the lungs. Subsequently, tumor cells in circulation may have been gradually eliminated by the immune system, and the clones settled in the lung would have progressed to the mediastinal lymph nodes. Hence, there was no intracranial tumor relapse, and we did not detect CTCs in the blood circulation.

The most common sites of metastasis of gliomas are the lungs, lymph nodes, bones, and liver.^6^, ^11^ Although metastasis occurred at common sites in the current case, to the best of our knowledge, this is the first reported case of simultaneous metastases in the lungs and mediastinal lymph nodes without intracranial relapse. The typical imaging manifestations misled our imaging experts at first. Accordingly, the detailed medical history and positive staining results for S100 and GFAP as well as negative results for TTF1 and P40 provided the accurate basis for the diagnosis of the metastatic disease.

Radical resection is the major treatment during the initial stage of LGG, while postoperative adjuvant radiotherapy and chemotherapy are the rational choice for HGGs.^12^, ^13^ However, there is no treatment consensus for metastatic gliomas. The median time to death was only 1 year from malignant transformation and 6 months from metastasis.^1^, ^14^, ^15^ The current patient underwent postoperative adjuvant radiotherapy and chemotherapy after the last craniotomy, and received palliative chemotherapy and molecular‐targeted therapy according to the results of the drug sensitivity test. In addition, he received ISB for the local lung metastasis after the metastasis was diagnosed, and the patient survived for 3 years after malignant transformation and 7 months after extracranial metastasis. Hence, he benefited from these treatments. Temozolomide is the standard chemotherapy drug for glioma, and external irradiation is relatively effective for HGG. Therefore, temozolomide as a systemic treatment combined with intensity‐modulated radiation therapy for the primary metastases in the lungs and mediastinal lymph nodes may have further improved the prognosis of this patient. In addition, cancer pain management of cancer patients is an important part of improving patients' quality of life. Studies have shown that patients with deep tumors have higher pain scores,^16^ postoperative analgesia is mainly administered with opioids.

In conclusion, the survival time of this glioma patient improved after transformation and metastasis. Detailed description will help us understand the biological behavior of glioma, but more studies are needed to confirm the complex mechanism of extracranial metastasis. Accordingly, the detailed medical history and tissue‐specific biomarkers are helpful for the diagnosis of extracranial metastasis of a glioma.

## AUTHORS CONTRIBUTIONS

Yunan Wang prepared the manuscript and the literature search. Hua Yang performed the histopathological, immunohistochemical, PCR, and CTC examinations. Jun Su performed the histopathological and immunohistochemical. Xiaobin Jian corrected and revised the manuscript. Peijie Li and Jianguo Zhou treated and found the patient. Wei Hu reviewed and edited the manuscript. All authors approved the final manuscript.

## CONFLICTS OF INTEREST STATEMENT

The authors declare no conflicts of interest.

## ETHICS STATEMENT

The patient was aware of this data recording and the informed consent was signed. This study was approved by the ethics committee of the Second Affiliated Hospital of Zunyi Medical University (No: KLL‐2021‐274).

## Supporting information


**Supplementary 1**. The enhanced T1WI MRI scan of the brain at different time points. A: The first relapse (September 2013); B: The first relapse postoperatively (September 2013); C: The second relapse postoperatively (December 2013).

**Supplementary 2**. The chest CT scan after I125 ion implantation (April 2017).

## Data Availability

The data are not available for public access because of patient privacy concerns but are available from the corresponding author on reasonable request. Graphical abstract figure created with Biorender. com.
