# Supplementary figures and images for: Transformation of a low‐grade glioma into a glioblastoma along with the development of lung and mediastinal lymph node metastases after repeated craniotomy: A case report
Source: Ibrain. 2023 Jul 6;10(3):385–9. doi: 10.1002/ibra.12119 (PMC11427796; doi:10.1002/ibra.12119)

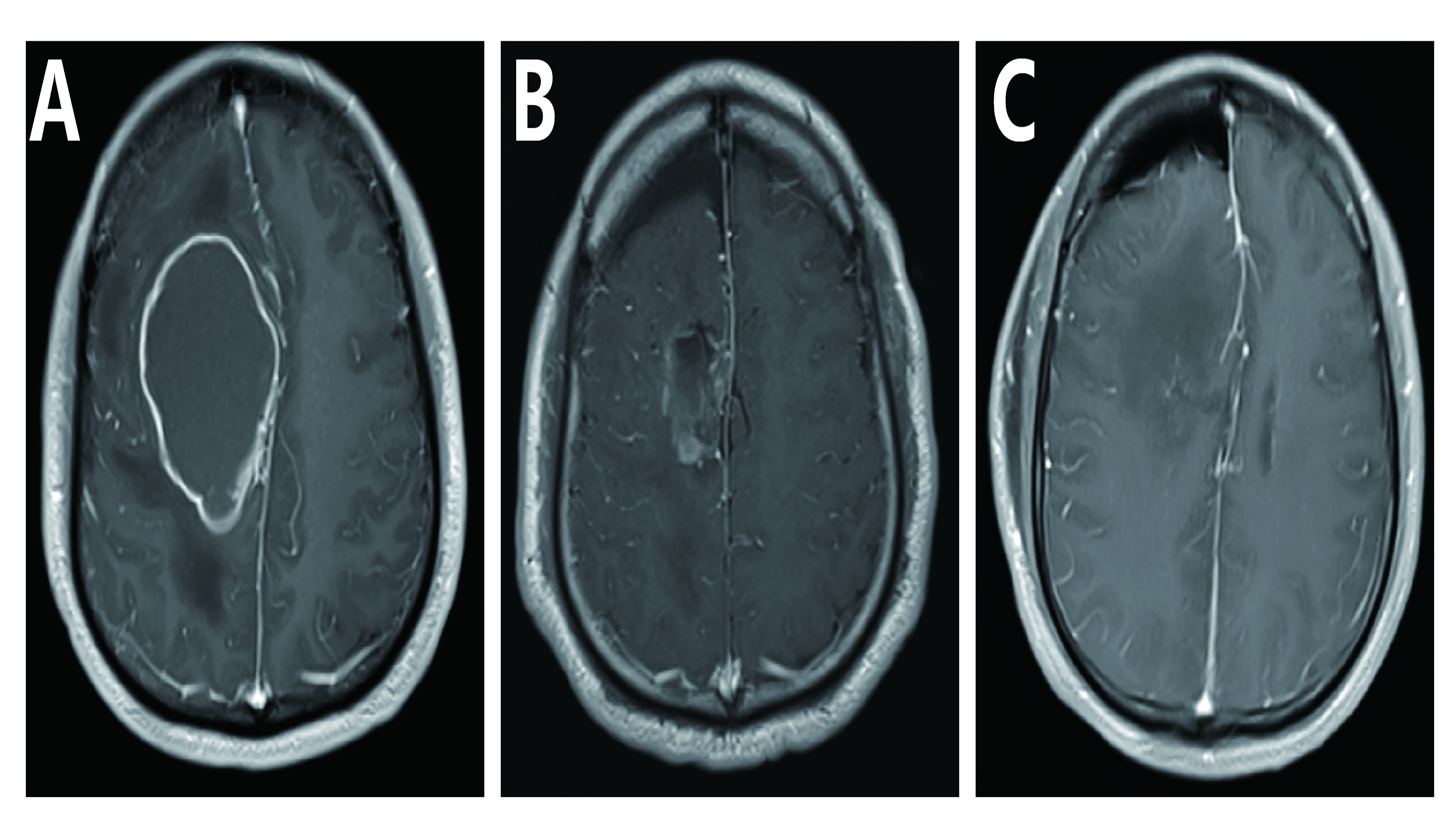

Supplement: Supplementary file 1 — Supplementary 1. The enhanced T1WI MRI scan of the brain at different time points. A: The first relapse (September 2013); B: The first relapse postoperatively (September 2013); C: The second relapse postoperatively (December 2013). [file IBRA-10-385-s002.tif]

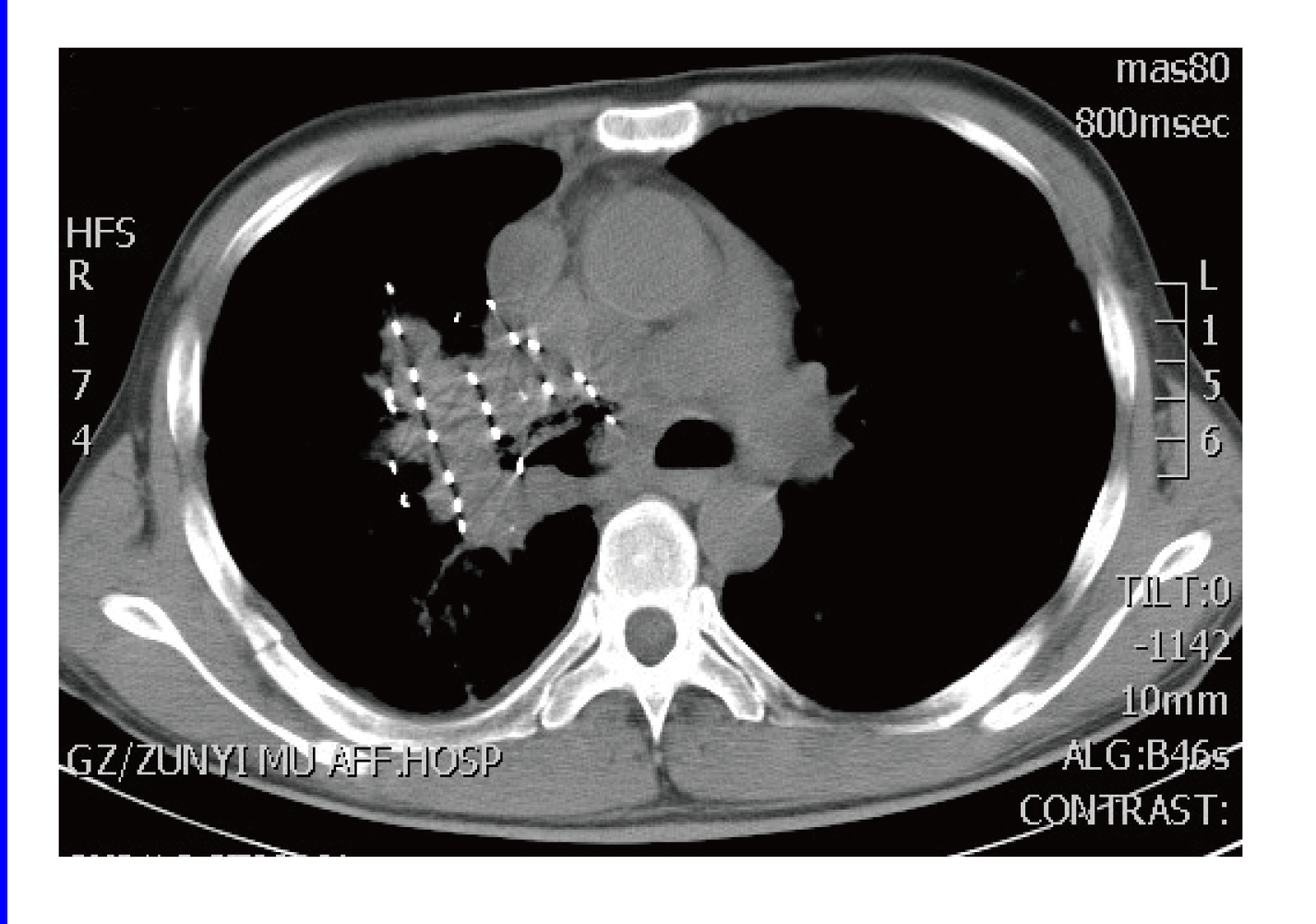

Supplement: Supplementary file 2 — Supplementary 2. The chest CT scan after I125 ion implantation (April 2017). [file IBRA-10-385-s001.tif]
